# Concomitant Cervicofacial Squamous Cell Carcinoma and Hepatocellular Carcinoma: A Rare Cadaveric Case Report

**DOI:** 10.7759/cureus.110236

**Published:** 2026-06-04

**Authors:** Hannah J Grimmett, Victoria Comfort, Megha Gupta, Kamal A Abouzaid, Ahmad Imam, Ehab M Ishteiwy

**Affiliations:** 1 Department of Anatomical Sciences, William Carey University College of Osteopathic Medicine, Hattiesburg, USA; 2 Department of Clinical Anatomy, William Carey University College of Osteopathic Medicine, Hattiesburg, USA; 3 Department of Internal Medicine, Al-Bayda Medical Centre (AMC) Omar Al-Mukhtar University, Al Bayda, LBY

**Keywords:** anatomy, case report, hepatocellular carcinoma, squamous cell carcinoma, synchronous multiple primary cancers

## Abstract

Multiple primary malignancies are uncommon, with the coexistence of cervicofacial squamous cell carcinoma (SCC) and hepatocellular carcinoma (HCC) being exceptionally rare. To our knowledge, very few cases describing concurrent SCC and HCC have been reported. This report describes the incidental cadaveric discovery of advanced SCC and HCC in the same individual and correlates gross anatomical findings with histopathologic evaluation. During a routine cadaveric dissection at William Carey University College of Osteopathic Medicine, a 64-year-old male cadaver was found to have extensive pathological changes involving the head and neck region and liver. Examination revealed prior mandibular resection with fixation hardware, tracheostomy placement, dense cervical fibrosis, and enlarged bilateral deep cervical lymph nodes. Histopathologic evaluation of cervical soft tissue demonstrated invasive SCC with infiltration through multiple tissue layers. Abdominal dissection revealed a markedly enlarged cirrhotic liver containing numerous nodules ranging from 0.5 to 5 cm, including a large caudate lobe mass compressing the portal triad. Histopathologic analysis confirmed multifocal HCC with portal vein invasion superimposed on advanced cirrhosis. This case highlights the rare synchronous occurrence of SCC and HCC identified incidentally during cadaveric dissection. It underscores the importance of thorough histopathologic evaluation in distinguishing multiple primary malignancies from metastatic disease and demonstrates the educational value of cadaveric dissection in correlating gross pathology with advanced malignancy.

## Introduction

Multiple primary malignancies are defined as the occurrence of two or more cancers, with no secondary relationship, occurring either concurrently or not, in a single patient [1]. Varying histological origins differentiate multiple primary malignant tumors and the metastasis of one primary cancer [2]. Although relatively uncommon, accounting for approximately 2-6% of all reported cancer cases, the incidence of multiple primary malignancies is thought to be increasing [1,2]. Most patients with a diagnosis of multiple primary cancers have no more than two, and, as the number of additional primary cancers increases, the incidence decreases [1].

Squamous cell carcinoma (SCC) is the most common oral cancer, accounting for approximately 90% of all oral malignancies, with the highest incidence reported in South and Southeast Asia [3,4]. Globally, intraoral SCCs contribute to 2.1% of cancer diagnoses and typically originate from nonkeratinized mucosa of the tongue and floor of the mouth, with the gingiva being the third most common intraoral subsite [5,6]. Chen et al. reported SCC in the gingiva in only 9.3% (572/6181) of the cases [7]. Additionally, SCC has a high predilection for lymph node metastasis and occurs more frequently in males [6,8]. Heavy alcohol and tobacco use are also directly correlated with developing intraoral SCC [6]. Clinically, gingival SCC initially appears as an intraoral swelling, mass, or ulceration. As the disease progresses, its clinical appearance can drastically vary; however, with progression, it is commonly described as an exophytic mass with a granular surface or as an ulcerative lesion. Due to its often asymptomatic, slow-growing nature, it is frequently misdiagnosed as a fibroma, chronic inflammatory enlargement, or pyogenic granuloma [8].

Liver cancer makes up approximately 5.6% of all cancers worldwide, making it the sixth most common malignancy, with a significantly higher incidence in males [5,9]. It is the third most common cause of cancer-related deaths following lung and stomach cancers [9]. Hepatocellular carcinoma (HCC) is the most common liver cancer, worldwide, making up between 75% and 85% of all liver cancer cases and is the leading cause of liver cancer-related deaths [10,11]. The primary risk factors for developing HCC include viral hepatitis (hepatitis B or C), excessive alcohol consumption, smoking, obesity, and diabetes in conjunction with oxidative stress and inflammation [12]. Of note, chronic viral hepatitis leads to the development of cirrhosis; cirrhotic livers have an increased proliferation of hepatocytes, which results in the growth of regenerative nodules and an increased risk of malignant transformation [11].

To our knowledge, the coexistence of SCC and HCC has only ever previously been reported in one patient in 1981 [13]. While there is no known correlation between the two primary cancers, it is important to note their concomitant existence, as it can help to guide the screening process for clinicians with high-risk patients. The present cadaveric case report describes the incidental discovery of coexisting SCC and HCC malignancies and highlights their potential clinical implications and manifestations.

## Case presentation

During the preparation of a prosection for pedagogical purposes at William Carey University College of Osteopathic Medicine, a formalin-fixed male cadaver, aged 64 at the time of death, was dissected. Two significant concomitant pathologies were identified: extensive surgical and pathological alterations in the head and neck region and a markedly enlarged, nodular liver. The cadaveric donor, a Caucasian male obtained through the University of Alabama Anatomical Gift Program, had a documented cause of death of cardiac arrhythmia; aside from this, no other clinical history was available.

Dissection of the head and neck region revealed prior surgical resection of the mandible, with placement of a metal fixation plate to maintain structural integrity. A tracheostomy tube had been placed antemortem, with a clearly visible midline incision site. Upon reflection of the cervical skin bilaterally, significant fibrotic adhesions were encountered, making dissection notably challenging. The skin flaps were reflected laterally and removed at the anterior border of the trapezius muscles.

**Figure 1:**
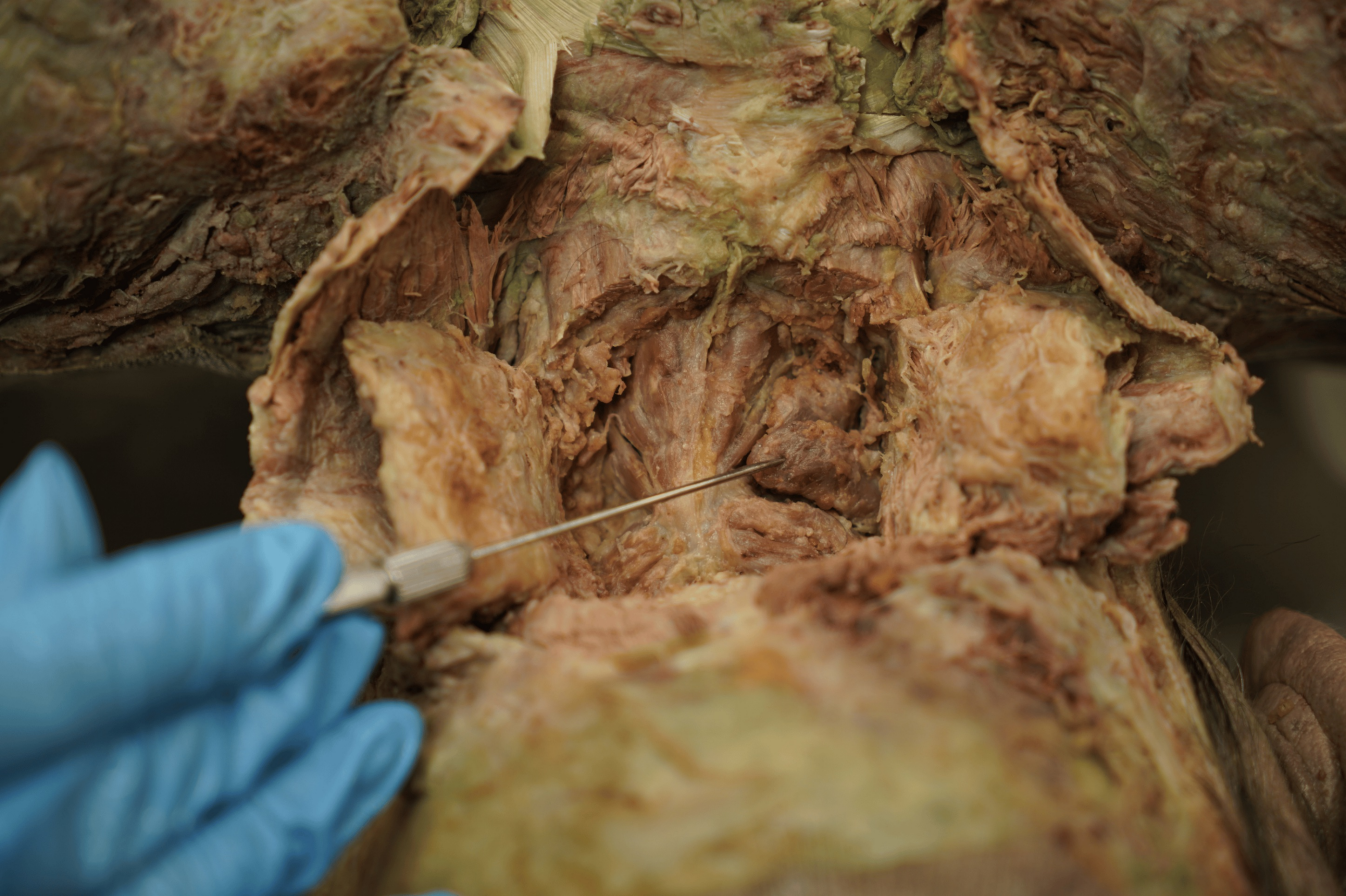
Enlarged lymph nodes within the suboccipital triangle

Further exploration demonstrated bilateral enlargement of the deep cervical lymph nodes and suboccipital lymph nodes, which were excised; these were not sent for histopathological examination, as the diagnosis of cervicofacial SCC was confirmed after the suboccipital area had been completely dissected (Figure 1).

**Figure 2:**
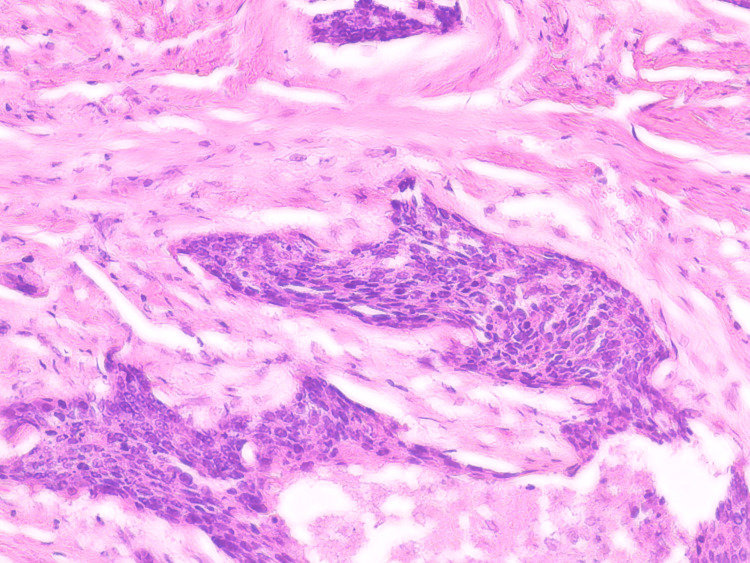
Histopathological examination of tissue specimen of the neck region showing atypical squamous cells indicative of squamous cell carcinoma Hematoxylin and eosin staining, magnification: ×40

As dissection proceeded into deeper tissue planes, extensive adhesions involving the fascia, musculature, and vascular structures of the anterior neck were observed, rendering anatomical planes difficult to distinguish. An excisional biopsy of the neck, including full-thickness skin and underlying soft tissue, was obtained for histopathological evaluation which revealed invasive SCC with infiltration across multiple tissue layers (Figure 2).

**Figure 3:**
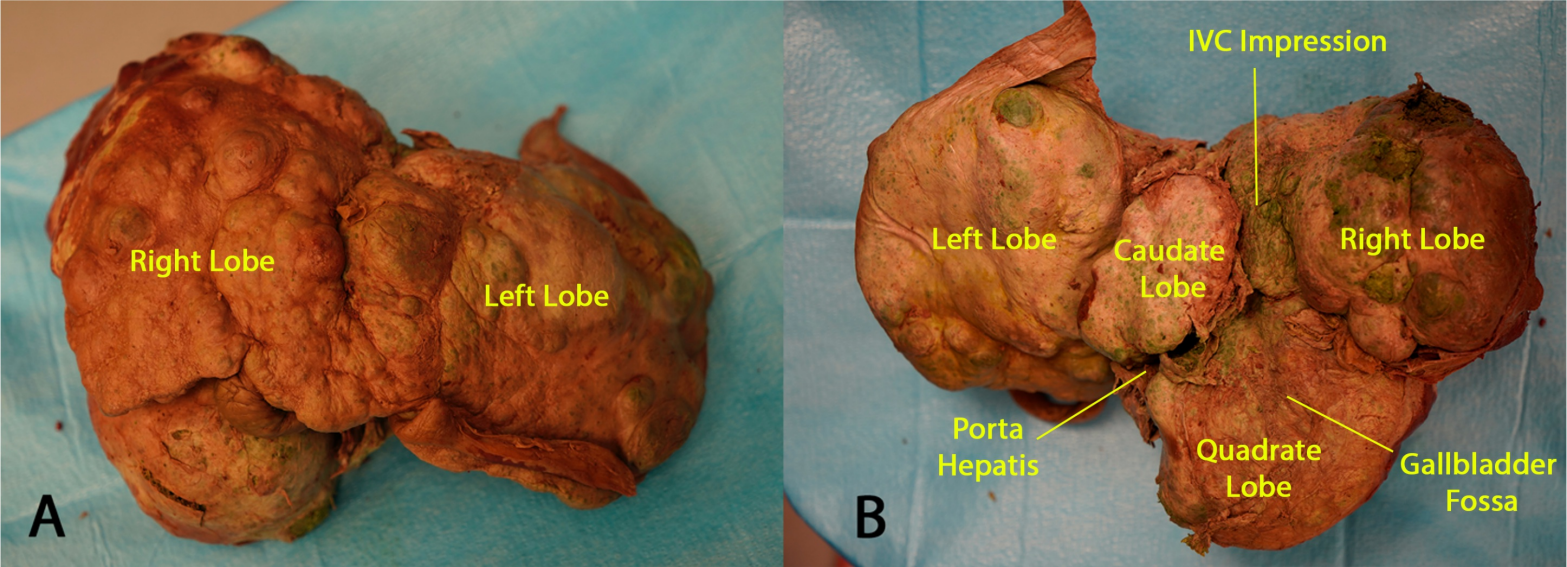
Gross view of the liver: (A) anterior view and (B) posterior view

During the dissection of the abdominal region, a remnant of a nasogastric tube was identified. The liver was exposed and appeared markedly enlarged and cirrhotic, with numerous well-defined, firm nodules distributed across all lobes. These nodules varied in size with diameters ranging from 0.5 cm to 5 cm and averaging approximately 1.5 cm (Figure 3A). The largest lesion was located in the caudate lobe, where it exerted compressive effects on the portal triad (Figure 3B).

**Figure 4:**
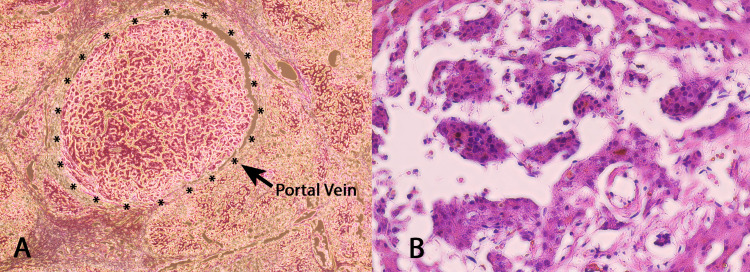
Histopathological examination of the liver specimen revealing cirrhosis and infiltration of the portal vein (A) with hepatocellular carcinoma (B) A: Hematoxylin and eosin staining, magnification: ×4. B: Hematoxylin and eosin staining, magnification: ×40

The liver was removed and measured 18.8 cm at its widest dimension, with a total weight of 2.64 kg. The caudate lobe mass was excised, and multiple additional nodules were biopsied and submitted for histopathological examination. Histopathologic analysis of the liver revealed advanced cirrhosis with superimposed HCC. The hepatic tumor showed invasion into the portal vein and was associated with areas of hepatic infarction (Figure 4).

## Discussion

The present case demonstrates the incidental cadaveric discovery of two advanced primary malignancies involving the head and neck region and the liver. Histopathologic evaluation confirmed invasive SCC within the cervical tissues and HCC arising in a cirrhotic liver with portal vein invasion. The coexistence of these malignancies is rare and provides a unique opportunity to correlate gross anatomical findings with advanced oncologic pathology.

It has been speculated that, because the focus of care is often directed toward the initial primary cancer diagnosis, the coexistence of a second primary malignancy may be overlooked [2]. Multiple primary malignancies have been associated with factors such as family history, immunologic or genetic deficits, and prolonged exposure to carcinogens [1]. In a review of 2,033 autopsies, Burke identified two primary malignancies in only 2.3% of cases. Reported combinations included scirrhous carcinoma of the stomach with alveolar carcinoma of the lung, adenocarcinoma of the rectum with hypernephroma of the kidney, and basal cell epithelioma of the lip with adenocarcinoma of the stomach [14]. Among these rare associations, the coexistence of SCC and HCC remains particularly uncommon [13].

The extensive fibrosis, tissue adhesions, mandibular resection, and cervical lymphadenopathy observed during dissection are consistent with advanced head and neck SCC and prior surgical intervention. Oral SCC accounts for more than 90% of malignant neoplasms of the oral cavity and commonly metastasizes to regional cervical lymph nodes [15]. Advanced gingival SCC is known to invade adjacent osseous structures, including the mandible, often necessitating surgical resection [16]. Involvement of the mandible classifies oral cavity SCC as an advanced-stage disease under the tumor, node, and metastasis (TNM) classification system [17]. The enlarged lymph nodes identified within the posterior cervical region may therefore reflect extensive regional dissemination. Although metastasis to level V lymph nodes is relatively uncommon in oral cavity SCC, its presence has been associated with advanced nodal disease [18].

The hepatic findings similarly reflected advanced malignancy. The markedly enlarged cirrhotic liver containing multiple nodules of varying sizes is characteristic of multifocal HCC arising in the setting of chronic liver disease. HCC, on average, accounts for approximately 80% of primary liver malignancies and commonly develops in patients with underlying cirrhosis. Histopathologic evidence of portal vein invasion further supports advanced-stage HCC, as vascular invasion is associated with aggressive tumor behavior and poor prognosis [19]. The gross findings of diffuse nodularity and the presence of a large caudate lobe mass compressing the portal triad correlated closely with the histopathologic findings of infiltrative carcinoma and hepatic infarction.

An important consideration in this case was whether the cervical lesion represented metastatic disease from the HCC. Although rare cases of oral metastasis from HCC have been reported, metastatic lesions typically retain hepatocellular morphology histologically [20]. In the present case, the cervical specimen demonstrated invasive SCC involving multiple tissue layers without hepatocellular features, supporting the interpretation of two distinct primary malignancies rather than metastatic spread. To our knowledge, only one prior report has described concomitant SCC and HCC in the same patient, further emphasizing the rarity of the present findings [13].

The precise etiologic relationship between these malignancies cannot be determined because of the limited clinical history available for the donor. However, tobacco and alcohol exposure have been identified as important risk factors for both SCC and HCC and may have contributed to the development of these tumors [6,12]. This case highlights the educational and pathological value of cadaveric dissection in identifying advanced disease processes and demonstrates the importance of correlating gross anatomical findings with histopathologic evaluation.

A limitation of this report is the absence of a comprehensive clinical history, including information regarding the donor's tobacco or alcohol use, viral hepatitis status, prior oncologic treatment, imaging findings, and timeline of disease progression. Because this was an incidental cadaveric discovery, correlation with antemortem clinical data, laboratory studies, and treatment records was not possible. Additionally, immunochemistry could not be performed. As a single cadaveric case report, these findings are inherently limited in generalizability and do not allow conclusions regarding causality or potential etiologic relationships between SCC and HCC. Despite these limitations, the case provides a rare pathological demonstration of concomitant SCC and HCC and highlights the value of cadaveric dissection and histopathologic evaluation in identifying advanced multifocal disease.

## Conclusions

This case highlights the rare coexistence of SCC and HCC identified incidentally in a cadaveric specimen. These findings underscore the diagnostic challenge of distinguishing multiple primary malignancies from metastatic disease. This report adds to the limited literature on concurrent SCC and HCC and emphasizes the need for further investigation into the mechanisms, risk factors, and clinical implications of multiple primary cancers. Recognition of such rare presentations is important for improving diagnostic accuracy and informing appropriate clinical management, as certain lifestyle habits are clinically established as being independent risk factors for the development of SCC and HCC.
